# Pulmonary Tuberculosis Complicating Interstitial Lung Disease in Systemic Sclerosis: A Case Report from a High TB Burden Country

**DOI:** 10.1155/crpu/8525357

**Published:** 2025-11-14

**Authors:** Donny Ardika Novananda, Garinda Alma Duta, Resti Yudhawati Meliana, Aris Sudarwoko

**Affiliations:** Pulmonology and Respiratory Medicine Department, Faculty of Medicine, Universitas Airlangga, RSUD Dr. Soetomo, Surabaya, Indonesia

## Abstract

Systemic sclerosis (SSc) is a chronic connective tissue disease marked by immune system dysfunction, vascular damage, and progressing fibrosis involving the skin and various internal organs. Interstitial lung disease (ILD) represents one of the leading contributors to illness and death in patients with SSc. The management becomes more complex when complicated by opportunistic infections such as tuberculosis (TB), particularly in endemic regions. We present the case of a 45-year-old woman with complaints of generalized weakness for one-week, intermittent fever, nausea and vomiting, and a weight loss of 5 kg over the previous 2 months. Clinical examination showed skin thickening and sclerodactyly, which then diagnosed as SSc-associated ILD (SSc-ILD). High-resolution computed tomography (HRCT) revealed reticular changes, ground-glass opacities, and traction bronchiectasis, indicating a nonspecific interstitial pneumonia (NSIP) pattern. During the course of treatment, the patient developed a persistent productive cough, weight loss, and fever. Pulmonary TB was confirmed through acid-fast bacilli smear and GeneXpert testing. Early recognition of dual pathology and coordinated care among rheumatology, pulmonology, and infectious disease teams are crucial for optimizing outcomes. This case underlines the need for vigilance in managing immunosuppressed patients in TB-endemic settings and contributes to the literature on SSc-ILD complicated by active TB.

## 1. Introduction

Connective tissue disease (CTD) described as a group of autoimmune conditions that are characterized by the immune system producing autoantibodies, leading to widespread organ involvement and damage. Systemic sclerosis (SSc), a major subtype of CTD, is marked by skin and internal organ fibrosis, microvascular damage, and immune system dysregulation resulting in the production of specific autoantibodies. SSc primarily occurs in women between the ages of 40 and 60 and exhibits diverse clinical features, such as Raynaud's phenomenon, digital ulcers, and pulmonary hypertension [1, 2].

Interstitial lung disease (ILD) is one of the most prevalent and possibly fatal pulmonary consequences of SSc, affecting up to 40% of patients. Approximately 10%–15% may experience progression to severe pulmonary fibrosis. ILD related to systemic sclerosis (SSc-ILD) may be the first sign of the disease and is frequently discovered during the initial assessment of individuals with suspected or confirmed SSc. A 2020 study in the United States reported the incidence of SSc and SSc-ILD at 16.4 and 1.2 per 100,000 person-years, with prevalence rates of 24.4 and 6.9 per 100,000, respectively [3, 4].

Tuberculosis (TB) is a chronic infectious disease mainly attributed to *Mycobacterium tuberculosis*. The World Health Organization (WHO) estimates that 10.6 million people were diagnosed with TB in 2021. Indonesia accounted for around 9.2% of all pulmonary TB cases worldwide, making it the nation with the second-highest number of cases. TB remains a leading cause of mortality among immunocompromised population, including those with autoimmune diseases such as CTDs [5–7].

Here, we present the case of a 45-year-old woman with SSc-ILD who subsequently developed pulmonary TB.

## 2. Case Presentation

A 45-year-old woman presented to the hospital in August 2022, with complaints of generalized weakness for 1 week, intermittent fever, nausea and vomiting, and a weight loss of 5 kg over the previous 2 months. She also reported reduced appetite and productive cough for 1 month. There was no history of night sweats, hemoptysis, or prior TB diagnosis. She denied smoking or alcohol use and had no known TB contacts in her home environment.

She had been previously diagnosed with CTD in December 2021, based on symptoms of joint stiffness and pain in both wrists and knees, amenorrhea, and progressive skin thickening. Since then, she had been on long-term immunosuppressive therapy, including azathioprine 50 mg/12 h and methylprednisolone 4 mg/12 h, along with folic acid 5 mg/24 h, lactate calcium 500 mg/24 h, and proton pump inhibitor—lansoprazole 40 mg/24 h, under rheumatology supervision. The immunosuppressive regimen was initiated to control autoimmune inflammation and prevent further fibrotic progression related to SSc-ILD. By the admission time, she had been on therapy for 8 months.

**Figure 1:**
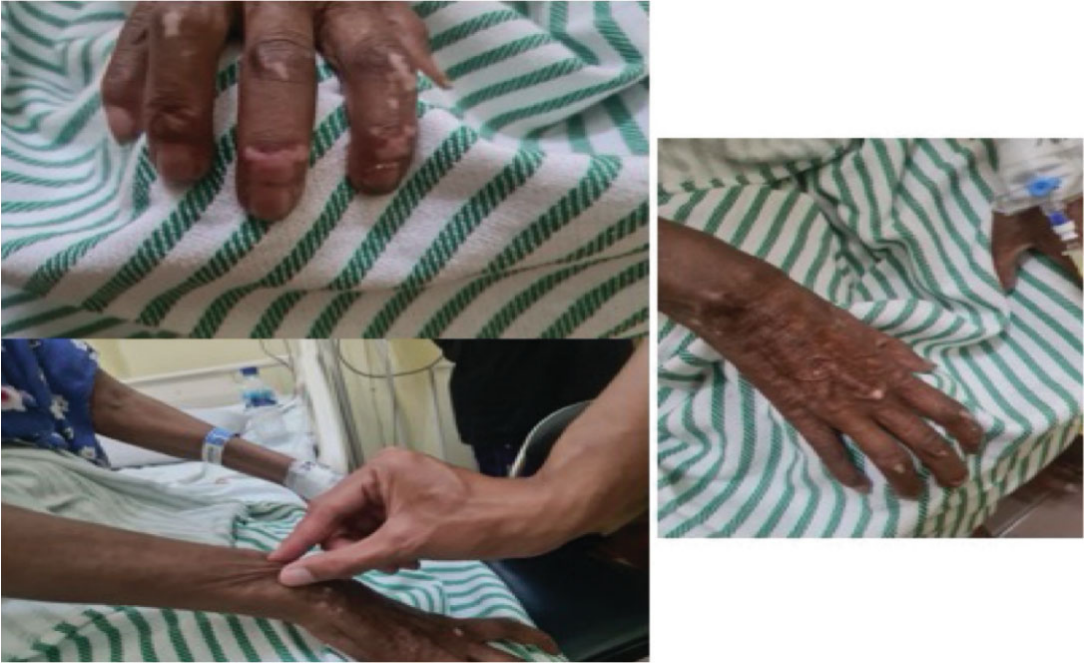
Sclerodactyly and skin thickening of both hands extending proximally, consistent with cutaneous manifestations of SSc.

Upon admission, her vital signs were found to be stable. Physical examination revealed generalized weakness, sclerodactyly, and skin thickening on both hands extending proximally (Figure 1). Velcro crackles were heard in the left lung field. Cardiovascular and abdominal examinations were unremarkable.

**Figure 2:**
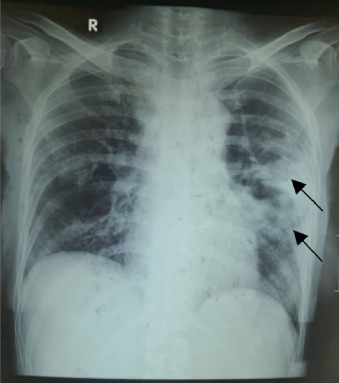
Thoracic radiograph showing left paracardial reticulogranular infiltrates, indicating pneumonia.

Initial laboratory tests showed normocytic normochromic anemia (Hb 7.4 g/dL), leukocytosis (14,400/*μ*L), thrombocytosis (894,000/*μ*L), hypoalbuminemia (2.87 g/dL), and hyponatremia (132 mmol/L). Laboratory examination also showed C3 106.3, C4 (38.05), and ANA test > 400. This indicates an autoimmune disease in the patient. Thoracic radiograph revealed left paracardial reticulogranular infiltrates, indicating pneumonia (Figure 2). Sputum examination revealed a positive result for acid-fast bacilli (AFB) (3+), and GeneXpert MTB/RIF test confirmed *Mycobacterium tuberculosis* with rifampicin sensitivity.

**Figure 3:**
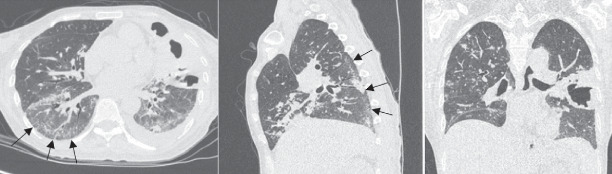
High-resolution CT scan of the chest showing reticular patterns in the posterobasal segments of both lower lobes with subpleural sparing, consistent with a NSIP pattern of ILD.

A high-resolution computed tomography (HRCT) scan on chest conducted in September 2022 demonstrated features consistent with a nonspecific interstitial pneumonia (NSIP) pattern, including bilateral ground-glass opacities, interlobular septal thickening, traction bronchiectasis, subpleural sparing, and fibrotic changes predominantly in the left lung (Figure 3). Additionally, the presence of tree-in-bud opacities and pulmonary nodules suggested concomitant pulmonary TB.

Based on the clinical, radiological, and laboratory findings, the patient was diagnosed with SSc-ILD with NSIP pattern and bacteriologically confirmed pulmonary TB. She was started on first-line anti-TB treatment (rifampicin, isoniazid, ethambutol, and pyrazinamide) and supportive therapy for ILD, including N-acetylcysteine and ibuprofen. Given the risk of exacerbating active infection, immunosuppressive agents (azathioprine and methylprednisolone) were temporarily withheld at the initiation of TB therapy. Immunosuppression was cautiously resumed after initial stabilization of the infection to prevent autoimmune reactivation and progression of pulmonary fibrosis, under close multidisciplinary supervision.

During hospitalization, her clinical condition gradually improved. Follow-up labs showed improvement in anemia and inflammatory markers, although hypoalbuminemia and hyponatremia persisted. She was discharged on September 2022, on a regimen of anti-TB drugs, ILD supportive treatment, and outpatient follow-up at the rheumatology and pulmonology clinics. However, the patient died at home on October 2022. No follow-up HRCT was performed before her demise.

## 3. Discussion

SSc, also known as scleroderma, is a chronic connective tissue that presents with a wide clinical spectrum, ranging from mild forms to severe disease involving internal organs and extensive skin fibrosis, often leading to significant disability [1]. The diagnosis of SSc in this patient was established using the 2013 European League Against Rheumatism (EULAR) classification criteria, which incorporates clinical and serologic findings (Table 1). Our patient fulfilled key diagnostic components, including proximal skin thickening extending beyond the metacarpophalangeal joints, the presence of sclerodactyly, and the presence of ILD. Based on the criteria, the patient scored 15, exceeding the 2013 EULAR threshold of ≥ 9 required for a SSc diagnosis [8].

The diagnosis of SSc-ILD involves integrating the clinical presentation, physical examination, lung function assessments, and radiographic imaging. Common symptoms include dyspnea, fatigue, and nonproductive cough, though early ILD may be asymptomatic. The clinical course of SSc-ILD is variable—most patients experience gradual pulmonary function decline, while others may show rapid progression, often indicated by increasing fibrosis on HRCT or worsening pulmonary function test results [3, 9].

According to the American Thoracic Society [10], the most frequent HRCT pattern seen in SSc-ILD is a NSIP, typically affecting the lower lobes and marked by ground-glass opacities, reticulation, and traction bronchiectasis. In this case, the patient's HRCT findings were consistent with NSIP, showing bilateral posterior basal reticulation, traction bronchiectasis, interlobular septal thickening, and ground-glass opacities with relative subpleural sparing—supporting the diagnosis of SSc-ILD.

The pathogenesis of SSc-ILD involves a multifaceted interaction among endothelial damage, immune dysregulation, and fibroblast activation. Initial vascular injury triggers endothelial activation, promoting vasoconstrictors like endothelin-1 and profibrotic mediators such as TGF-*β*. Immune cells, notably Th2 cells and macrophages, contribute to fibrosis by releasing IL-4 and IL-13, which stimulate fibroblast-driven extracellular matrix deposition. Autoantibodies, particularly anti-topoisomerase I, are linked to increased ILD risk and severity, although this patient's autoantibody status remained partially undefined [11].

Immunosuppressive therapy is the mainstay of SSc-ILD management. Agents such as cyclophosphamide and mycophenolate mofetil (MMF) have demonstrated efficacy in slowing disease progression in randomized clinical trials [9]. Azathioprine is often used in milder or maintenance settings. While antifibrotic agents like nintedanib have shown benefit in idiopathic pulmonary fibrosis (IPF) and were recently approved for SSc-ILD, their use remains limited because of access, cost, and disease-specific considerations [12, 13]. In this case, nintedanib was not initiated, given the primary emphasis on immunosuppression.

An important clinical challenge in this case was the development of bacteriologically confirmed pulmonary TB during the course of SSc-ILD. Chronic ILDs can compromise local host defenses, thereby increasing susceptibility to TB infection. Moreover, patients with SSc are at heightened risk because of underlying immune dysregulation and the use of immunosuppressive therapies [14]. Diagnosing TB in patients with SSc-ILD can also be particularly challenging. Radiological findings such as reticular opacities or honeycomb patterns may mimic pulmonary TB, leading to potential diagnostic confusion. Therefore, in immunosuppressed patients or those with additional risk factors such as diabetes mellitus, TB screening with GeneXpert or sputum culture is recommended to ensure diagnostic accuracy [15].

The diagnosis of TB was confirmed based on persistent productive cough, constitutional symptoms, and positive AFB smear and GeneXpert testing. The dual pathology of SSc-ILD and TB presented a clinical dilemma, as immunosuppressive therapy is necessary to control SSc-ILD but may worsen TB infection. Coordination between pulmonology, rheumatology, and infectious disease teams was essential to balance risks and benefits [16].

Immunosuppressive therapy plays a dual role in the management of SSc-ILD. While agents such as corticosteroids or azathioprine are essential to control autoimmune inflammation and prevent fibrotic progression, they also compromise host immunity, thereby increasing susceptibility to opportunistic infections, particularly TB. Patients with SSc already exhibit immune dysregulation, and additional immunosuppression further predisposes them to reactivation of latent TB infection or the development of new infection. This interplay creates a therapeutic dilemma, as highlighted in previous reports, where immunosuppressed patients with SSc-ILD demonstrated higher rates of infectious complications, including TB. Therefore, clinicians must balance the need for immunosuppressive control of SSc-ILD against the heightened infection risk, and consider pretreatment TB screening, especially in high-burden regions [17, 18].

In this case, the patient was initiated on first-line anti-TB therapy (ATT), while immunosuppression was temporarily withheld to prevent the presentation of nontypical diseases and a higher risk of treatment failure. The immunosuppressive therapy was later cautiously resumed after initial TB stabilization. The decision to resume the therapy was guided by the need to prevent progression of SSc-related pulmonary involvement once infection control was achieved. Careful timing is critical, as premature reintroduction may exacerbate TB, whereas prolonged discontinuation can lead to worsening fibrosis and decline in lung function [19, 20].

Although the patient's condition had temporarily stabilized at discharge, she later passed away. The risk of mortality in patients with SSc-ILD increases with some comorbidities, including TB infection, which may further worsen the prognosis by aggravating pulmonary inflammation and compromising respiratory function. Pulmonary complications are the primary contributor to mortality among immunosuppressed individuals, particularly SSc. Immunosuppressive therapy suppresses TNF-*α* and interferon-*γ*–mediated immune responses, impairing granuloma integrity and allowing reactivation or spread of *Mycobacterium tuberculosis*. Furthermore, respiratory failure may have resulted from the combined effects of TB and pre-existing ILD. Tuberculous inflammation superimposed on fibrotic lungs could have triggered acute ILD exacerbation or acute respiratory distress syndrome, both associated with high mortality [15, 17, 21].

## 4. Conclusion

This case illustrates the challenging clinical course of SSc-ILD complicated by infection of TB, emphasizing the need for early recognition, multidisciplinary collaboration, and careful monitoring—especially when managing immunosuppression in TB-endemic areas. Despite initial stabilization, the patient's deterioration reflects the high mortality risk from pulmonary involvement in SSc. Routine TB screening before and during immunosuppressive therapy in autoimmune patients is crucial, particularly in high-prevalence regions, to enable early detection and reduce complications.

## Figures and Tables

**Table 1:** 2013 EULAR classification criteria for systemic sclerosis and patient scoring.

**Item**	**Subitem**	**Score**	**Patient's score**
Skin thickening of the fingers of both hands extending proximal to the metacarpophalangeal joints	—	9	9
Skin thickening of the fingers of both hands extending proximal to the metacarpophalangeal joints	Puffy fingers	2	—
	Sclerodactyly	4	4
Fingertip lesions (only highest score is counted)	Digital ulcers	2	—
	Fingertip pitting scars	3	—
Telangiectasia	—	2	—
Abnormal nailfold capillaries	—	2	—
Pulmonary arterial hypertension and/or interstitial lung disease (maximum score 2)	Pulmonary arterial hypertension (SSc-PAH)	2	—
	Interstitial lung disease (SSc-ILD)	2	2
Raynaud's phenomenon (SSc-RP)	—	3	—
Systemic sclerosis-related autoantibodies (maximum score 3	Anticentromere	3	—
	Anti-topoisomerase I		—
	Anti-RNA polymerase III		—

## Data Availability

Data sharing not applicable to this article as no datasets were generated or analyzed during the current study.
